# Health Behaviors of Colombian First-Semester University Students in Association with Behaviors of Close Social Ties, Living Arrangement, and Time Spent with Peers

**DOI:** 10.3390/ijerph20075370

**Published:** 2023-04-03

**Authors:** Chrys Gesualdo, Martin Pinquart, Ana Chamorro Coneo, Moises Mebarak Chams

**Affiliations:** 1 Department of Psychology, Philipps-University, 35037 Marburg, Germany; 2 Department of Psychology, Universidad del Norte, Barranquilla 081007, Colombia

**Keywords:** first-semester freshmen, food consumption, physical activity, alcohol use, Colombia

## Abstract

Objective: in Colombia, many first-year university students consume unhealthy food, are physically inactive, and drink regularly, which can be associated with the behavior of social ties, living with social ties, and time with peers. The present cross-sectional study assessed the association between health behaviors of first-semester students and these factors. Method: *N* = 189 (*M_age_* = 18.79; *SD* = 1.07; female = 68.8%) first-semester students in Colombia completed an online questionnaire investigating current and expected health behaviors as well as influencing factors. ANCOVAs, bivariate correlations, moderation analyses, and hierarchical regressions were used to analyze the data. Results: expected food consumption (stronger among participants who live with parents) as well as current and expected heavy drinking and binge drinking (stronger among participants who do not live with parents) were significantly correlated to the respective parental behavior. Current and expected drinking was significantly correlated to partners’ drinking. Expected physical activity was correlated with peers’ physical activity. Partners’ attempts to encourage drinking moderated the association between participants’ current and expected drinking with partners’ drinking. Time spent with peers was related to heavy drinking and engaging in more physical activity. Conclusion: in Colombia, parents appear to play a significant role in their offspring’s health behaviors during their first semester at university, particularly regarding food consumption and alcohol use. Partners’ drinking and time spent with peers are strongly related to heavy drinking.

## 1. Introduction

During their first semester at university, many students tend to engage in health-risk behaviors such as the consumption of unhealthy food, physical inactivity, and problematic alcohol use [1]. In Colombia, an alarmingly high percentage of university students are overweight or obese and the prevalence of these conditions have been associated with inappropriate nutrition (e.g., high in sugar and fat) and sedentary behavior [2]. Consistent with this notion, a recent study among first-semester Colombian freshmen found that a high percentage of students consume unhealthy food items and that most students reported not participating in sports [3]. If not corrected, these behaviors may lead to hypertension, type 2 diabetes, and osteoarthritis [4]. Furthermore, many first-year students in Colombia report consuming alcohol on a weekly basis as they are often exposed to opportunities for the initiation and increase of alcohol use [3], highlighting the need to significantly reduce this behavior to prevent negative health effects. If problematic alcohol use among freshmen is not prevented or addressed, it may lead to an early decline of health as well as to academic, familial, and social difficulties [5]. Moreover, previous experiences with alcohol as well as expected effects from drinking have been associated with present alcohol use among Colombian undergraduate students [6]. In addition, Mora-Ríos and Natera [7] reported that positive alcohol expectancies were related to greater alcohol intake among Mexican college students. As such, students are at high risk of behaving and expecting to behave in risky manners during their first semester at university [8].

### 1.1. Influences on Health Behaviors and Expectations

Current and expected health behaviors (food consumption, physical activity, and alcohol use) among first-semester students have been associated to the health behaviors of close social ties, to their efforts to motivate behavior, and to co-residence with close social ties. For instance, current and expected physical activity has been previously associated to the related behavior of parents, partners, and peers, while current and expected eating has been associated to peers’ eating [9]. In addition, parental influence in consuming alcohol as well as parents’ own drinking patterns was associated with the initiation and continued use of alcohol among a sample of Colombian university students [10]. Moreover, peer influence to consume alcohol as well as perceived drinking in the peer group was associated with alcohol use among Colombian students as alcohol is often seen as a socialization agent that assists in the establishment of peer relations [10]. Arango-Paternina and colleagues [11] found that Colombian female students whose peers were not physically active were more likely to not be physically active themselves compared to students whose peers were physically active. Furthermore, Troncoso and Amaya [12] found that parents and peers significantly influence Latin American students’ food consumption habits, with parents showing stronger influence. Thus, the health behavior of close social ties and their efforts to motivate behavior may have an effect on health behaviors and expectations among university students, although the degree of each social tie’s influence may differ based on further factors, such as living arrangement [13].

The living arrangement of first-year students has been shown to influence their health behaviors and expectations. For instance, German first-semester students were more likely to engage in alcohol use with their partners and peers in the case of co-residence with these social ties [9]. Mixed results exist regarding whether leaving the parental home to attend university is related to increased health-risk behaviors among students. Some findings suggest that students who moved out of their parents’ home show more unhealthy behaviors than those who did not move out [14]. A similar finding among Dutch university students suggests that university students that do not live with their parents show a less unfavorable health behavior profile than students that live with peers, with their partner, or alone [15]. Moreover, Deliens and colleagues [16] found that students that live with their parents perceive their living arrangement as a barrier against risky health behaviors, so that students expect to behave in a healthier manner while living with their parents. Conversely, Betancourth-Zambrano and colleagues [10] reported that Colombian students that live with their parents show the highest rates of alcohol use. It is not clear whether similar results would be found in Colombia regarding food consumption and physical activity. As such, mixed findings regarding whether living with parents is related to more healthy or unhealthy behaviors may be related to differences in parental permissiveness, monitoring, and modeling of (un)healthy behaviors [17].

Lastly, time spent with peers may also influence students’ food consumption, physical activity, alcohol use, and respective behavioral expectations. For instance, first-semester students in Germany who reported spending more time with their peers were more likely to consume higher amounts of alcohol [9]. Similarly, Colombian university students appear to consume alcohol more often while being with their peers [10]. Accordingly, time spent with peers is an important predictor of health behaviors among college students.

### 1.2. Hypotheses Development and Hypotheses

Health risk behaviors among first-semester students has mainly been investigated among Western samples. Moreover, the number of related studies conducted in Latin American countries are small despite the evident need to further investigate health-risk behaviors among this population, as well as factors that facilitate these behaviors. Furthermore, scant non-Western studies investigated food consumption, physical activity, and alcohol use among first-semester students simultaneously while also investigating contributing factors. Based on the aforementioned situation, investigating these matters will allow the further understanding of health behaviors and related expectations among Colombian first-semester students specifically, and will provide insights related to factors that influence students’ behaviors and expectations (i.e., which social ties are most strongly associated to students’ health behaviors). The present study assessed whether differences between current and expected future food consumption, physical activity, and alcohol use exist among first-semester Colombian students based on their living arrangement and the health behaviors of their close social ties (i.e., parents, partners, or peers). Hypothesis 1 states that students who moved out of the parental home expect stronger increases of unhealthy behaviors than students who did not move out. Hypothesis 2 posits that students’ health behaviors and related expectations are correlated to the reported health behaviors of their close social ties. Hypothesis 3 suggests that the correlations addressed in Hypothesis 2 are stronger in the case of co-residence with the social tie because living with social ties provides more opportunities for being influenced. Furthermore, we assessed whether students’ health behaviors are related to the efforts of social ties to motivate behaviors and to time spent with their peers. Hypothesis 4 postulates that social ties’ attempts to motivate health behaviors moderate the association assumed in Hypothesis 2. Finally, Hypothesis 5 proposes that students that spend more time with their peers behave in unhealthier ways than students that spend less time with their peers or who still live with their parents.

## 2. Method

A quantitative research design was used for the present study. The study was conducted in accordance with the Declaration of Helsinki, and the protocol was approved by the Ethics Committee at Universidad del Norte, Colombia (protocol code: 269, date of approval: 30 June 2022) and at the University of Marburg, Germany (protocol code: 2020-79k, date of approval: 9 May 2022). Recruiting emails were sent to first-semester Colombian university students of at least 18 years of age during the first week of the semester (end of July 2022). All subjects gave their informed consent for inclusion before they participated in the study; were fully informed about the purpose of the research; about how their data would be used; about any risks associated; and their anonymity was assured. In order to measure students’ behaviors early in the first semester before being strongly influenced by the college environment, data collection took place from the end of July to the end of August 2022. Students received a link directing them to the study and completed the questionnaire online. Participants were asked to indicate whether they wanted to participate in a gift card raffle as compensation for the time invested. Translation and back translation were used for conversion of the English language instruments into Spanish [18].

### 2.1. Measures

#### 2.1.1. Sociodemographic and Environmental Characteristics

Items assessed age, sex (male, female, non-binary), whether the participant has a partner or not, whether students moved out of the parental home or not, current living situation (i.e., with parents, shared apartment, student dorm, with partner, other living arrangements), hours per day spent with peers, and whether the hometown is in Colombia or abroad (i.e., specific ethnic background was not assessed to protect participant anonymity).

#### 2.1.2. Healthy Eating

The unhealthy food consumption scale of the Centers for Disease Control and Prevention’s National College Health Risk Behavior Survey (NCHRBS) [19] was administered to assess how many times a day during the past month participants consumed unhealthier (i.e., hamburgers, hot dogs/sausages, fried potatoes/chips, cookies/doughnuts/cake) food items. Two items following the NCHRBS’s format were designed and included to assess additional unhealthier food typically consumed by university students (i.e., pizza, sweets, and chocolate). Thus, we administered five items to assess the consumption of unhealthier food. A Likert scale response format of 1 = 0 times a day, 2 = 1 time a day, 3 = 2 times a day, and 4 = 3 or more times a day was used. In addition, we included rephrased forms of the five items to investigate expected daily unhealthy food consumption during the first semester. We found an internal consistency of *α* = 0.72 for present behavior and of *α* = 0.87 for expected future behavior, indicating good reliability [20].

#### 2.1.3. Physical Activity

The NCHRBS [19] was also administered to assess how many times a week in the last month participants performed physical activity including stretching, strengthening exercises, and walking or cycling. The four items had a Likert scale response format ranging from 1 = 0 to 8 = 7 times a week. Responses were inverted so that higher scores reflect lower physical activity levels. Rephrased forms of the four items were also included to investigate expected weekly physical activity during the first semester. The scale showed a good/excellent internal consistency of *α* = 0.80 (actual behavior) and of *α* = 0.90 (expected future behavior).

#### 2.1.4. Alcohol Use

We included three items from the Alcohol Use Disorders Identification Test (AUDIT) [21] to assess: (a) on how many days of the past month did the participant consume alcohol; (b) how many standard drinks did the participant drink on a drinking occasion; and c) how often the participant drank five (for males), four (for females), or more standard drinks on a drinking occasion. The first two items had a free input response format and responses for the two items were multiplied to obtain the number of drinks consumed by participants in a month. The third item has a Likert scale response format ranging from 1 = never, 2 = less than monthly, 3 = monthly, to 4 = weekly. If participants reported consuming five (for males), four (for females), or more drinks on a drinking occasion they were considered binge drinkers. Rephrased formats of the three items assessing expected drinking during the first semester were also included.

#### 2.1.5. Health Behaviors of Social Ties

Nine items (i.e., three per social tie assessing each of the three health behaviors) were developed for our study to investigate participants’ perceptions regarding the frequency in which their close social ties (i.e., parental dyad, partner, and peer group) consume healthy food, perform physical activity, and drink alcohol. Each health behavior was assessed with one item per social tie, and only participants who reported having a partner completed the items assessing the partner’s behaviors. Response options were in a Likert scale format ranging from 1 = very often, 2 = often, 3 = rarely, to 4 = never. Higher scores were coded to reflect unhealthier behavior.

#### 2.1.6. Social Ties’ Efforts to Encourage Health Risk Behaviors

Nine items (i.e., three per social tie assessing each of the three health behaviors) were developed for our study to investigate participants’ perceptions regarding how often each social tie (i.e., parental dyad, partner, and peer group) motivates them to eat unhealthy food, to be physically inactive, and to drink alcohol. Only participants who reported having a partner completed the three items assessing the partner’s motivation efforts. Responses had a Likert scale format ranging from 1 = never, 2 = rarely, 3 = often, to 4 = very often.

### 2.2. Statistical Analysis

IBM SPSS Statistics version 27 was used to analyze the results. A minimum sample size of 180 participants was need as indicated by a power analysis to identify small effects with 80% power at an alpha level of 0.05. To examine baseline group differences (moved out of the parental home, age, sex, where the hometown is), independent *t*-tests and chi^2^ analyses were conducted. The first hypothesis was analyzed with analyses of covariance, in which we included differences between current and expected behavior as dependent variables with age and where the hometown is as covariates. The second hypothesis was tested using bivariate correlations, while the third and fourth hypotheses were investigated with moderation analyses using multiple regression. The fifth hypothesis was assessed by computing hierarchical regressions.

## 3. Results

### 3.1. Sociodemographic Characteristics

A total of N = 193 students participated in our study. Four respondents were excluded because of extreme scores of >2 standard deviations above the group mean. This resulted in a total sample of N = 189 (M_age_ = 18.79; SD = 1.07); female = 68.8%; male = 31.2%; see Table S1). Moreover, only 32.3% reported having a partner. Most of the sample (95.2%) reported that their hometown is in Colombia and that they did not move out of their parents’ home (66.7%). Age was slightly lower among participants who moved out (M = 18.57; SD = 0.91) compared to participants who did not move out of the parental home (M = 18.90; SD = 1.13); a mean difference of −0.33 (95% CI, −0.65 to −0.01) years was found, t(187) = −1.98, *p* = 0.05. No significant sex differences nor differences regarding whether the hometown is in Colombia or abroad were found among those that moved out and those who did not. Lastly, 63.5% reported co-residence with their parents, 9% lived in a shared apartment, 3.2% lived in a student dorm, 3.7% lived with their partner, while 13.8% reported other living arrangements.

### 3.2. Health Behaviors and Living Arrangement

As small age differences were found between participants who moved out and those who did not, age was included as a covariate in the analysis. No significant differences were found between participants who moved out of the parental home and those who did not regarding discrepancy between current and expected future food consumption F(1,189) = 0.01, *p* = 0.92, physical activity F(1,189) = 0.02, *p* = 0.89, number of drinks consumed per month F(1,189) = 2.78, *p* = 0.10, and binge drinking F(1,189) = 0.29, *p* = 0.59. Thus, our first hypothesis was not supported. As the observed lack of group differences may indicate that movers have already adapted their health behaviors to the new environment, we further tested whether the mean scores of present unhealthy behaviors differed between those who moved out and those who did not move out. Participants who moved showed less physical activity (M = 23.37, SD = 6.69) and more binge drinking (M = 2.13, SD = 1.06) than participants who did not move out (for physical activity M = 21.17, SD = 6.86, t(187) = 2.09, *p* = 0.04; for binge drinking (M = 1.77, SD = 0.88; t(187) = 2.46, *p* = 0.02). No significant differences were found regarding food consumption (moved out M = 19.84, SD = 4.30; did not move out M = 20.33, SD = 4.02; t(187) = −0.76, *p* = 0.45) and heavy drinking (moved out M = 6.14, SD = 8.61; did not move out M = 5.04, SD = 7.06; t(187) = 0.94, *p* = 0.35).

### 3.3. Social Ties’ Health Behaviors

The expected food consumption pattern of participants was significantly correlated to the food consumption patterns of their parents (r = 0.18, *p* = 0.01). Moreover, participants’ current and expected number of drinks consumed per month (r = 0.22, *p* = 0.01 and r = 0.23, *p* = 0.01, respectively) as well as current and expected binge drinking (r = 0.34, *p* = 0.001 and r = 0.27, *p* = 0.001, respectively) were significantly correlated with parental alcohol use. However, participants’ current food consumption (r = 0.07, *p* = 0.31) and current (r = 0.12, *p* = 0.11) and expected (r = 0.07, *p* = 0.33) physical activity were not significantly associated with the respective parental behaviors. Regarding the partner, perceived alcohol use of partners correlated with participants’ current number of drinks consumed per month (r = 0.34, *p* = 0.01) as well as their current and expected binge drinking (r = 0.54, *p* = 0.001 and r = 0.34, *p* = 0.01, respectively). No significant associations regarding partners’ respective behaviors and participants’ current and expected food consumption (r = 0.07, *p* = 0.62 and r = 0.05, *p* = 0.71, respectively), current and expected physical activity (r = 0.14, *p* = 0.27 and r = 0.12, *p* = 0.34, respectively), and expected number of drinks consumed per month (r = 0.20, *p* = 0.12) were found. Lastly, regarding peers, a significant correlation was found between participants’ expected physical activity and their peers’ physical activity (r = 0.20, *p* = 0.01). No significant associations of peers’ respective behaviors and participants’ current and expected food consumption (r = 0.11, *p* = 0.13 and r = 0.09, *p* = 0.20 respectively), current and expected number of drinks consumed per month (r = 0.11, *p* = 0.14 and r = 0.06, *p* = 0.40 respectively), current and expected binge drinking (r = 0.12, *p* = 0.11 and r = 0.13, *p* = 0.08 respectively), as well as current physical (r = 0.10, *p* = 0.19) activity were found. Therefore, our second hypothesis was supported regarding parents in relation to food consumption and alcohol use, regarding partners in relation to alcohol use, and regarding peers in relation to physical activity.

### 3.4. Co-Residence with Social Ties

As most participants reported living with their parents, we did not analyze moderating effects of living with peers and partners due to restricted statistical power as results would not provide valuable information. Results for co-residence with parents are presented in Table 1. Co-residence with parents moderated the relationship between participants’ expected food consumption, current number of drinks consumed per month, as well as current and expected binge drinking and the respective parental behavior. No significant moderation effects were found regarding current food consumption, current and expected physical activity, and expected number of drinks consumed per month. Further analyses showed that the association of participant-expected food consumption and parental food consumption was stronger among participants who live with their parents (β = 0.96; *p* = 0.04) than among participants who do not live with their parents (β = 0.75; *p* = 0.13). The association between participant and parental alcohol use was stronger among participants who do not live with their parents (β’s ranged from 0.60 to 0.38 and p’s ranged from 0.01 to 0.001) than among participants who live with their parents (β’s ranged from 0.14 to 0.08 and p’s ranged from 0.36 to 0.11). Thus, support for the third hypothesis was only found regarding co-residence with parents and students’ expected food consumption patterns.

### 3.5. Social Ties’ Efforts to Encourage Health-Risk Behaviors

Efforts from the partner to consume alcohol moderated the relationship of participants’ binge drinking behavior and expectations with partners’ alcohol use patterns (see Table 2). Follow-up analyses indicated that the association between participant current and expected binge drinking and partners’ alcohol use was stronger among participants whose partner frequently encourages drinking (β’s ranged from 0.40 to 0.30 and p’s ranged from 0.02 to 0.01) than among participants whose partner does not frequently encourage drinking (β’s ranged from 0.10 to 0.05 and p’s ranged from 0.51 to 0.92). Significant moderating effects of social ties’ encouragement for other kinds of unhealthy behavior were not found. Accordingly, the fourth hypothesis was only supported regarding partners’ encouragement to drink alcohol.

### 3.6. Time Spent with Peers

The fifth hypothesis was only supported regarding time spent with peers and a higher number of drinks consumed by participants per month (Table 3). Results for binge drinking were in the expected direction but did not reach the 5% significance level. Results also indicate that students who spend more time with their peers were more likely to engage in physical activity.

## 4. Discussion

The present study assessed whether differences in current and expected health behaviors exist among first-semester Colombian university students based on their living arrangement, the health behaviors of their close social ties, their social ties’ efforts to motivate unhealthy behaviors, and time spent with their peers. In general, we found that some current as well some expected behaviors were correlated to respective behaviors among social ties. Moreover, encouragement from partners to consume alcohol moderated the association between participants’ and partners’ drinking behaviors. Furthermore, time spent with peers was associated with participants’ behaviors. A summary of the main findings is presented below followed by a detailed discussion.

First, students who moved out of the parental home did not expect larger changes in their health behaviors than those who still live with their parents. As previous studies posit that students are expected to behave in unhealthier ways after moving out of the parental home to attend university [14,22,23], our results indicate that participants who moved out have already increased unhealthy behaviors by the time of our assessment as the mean scores of present physical inactivity and binge drinking were higher among those who moved out than among those who did not. As they have already adapted their health behaviors to their new environment, they need not expect strong and further changes across the first semester. In addition, as more than two-thirds of the respondents still lived with their parents, statistical power was limited for finding small or very small effects of leaving the parental home.

Second, students’ health behaviors and expectations were correlated to parental food consumption and alcohol use, and the correlation between students’ expected food consumption patterns and parental food consumption was stronger in the case of co-residence with parents. Students seem to have (and expect to have) food consumption patterns as well as drinking patterns that tend to be similar to the related parental behavior. Latin American students appraise their food consumption habits as having originated in their childhood and being established by their parents [12]. Thus, parental food consumption habits appear to transfer to their offspring who may have preserved these habits even after reaching adulthood [12]. The finding regarding participant behavioral similarity to parental alcohol use may be explained by Betancourth-Zambrano and colleagues’ [10] finding indicating that, in Colombia, parental approval of their offspring’s alcohol use fosters drinking among youth. In Colombia, early alcohol consumption through offerings from the family is common [17,24]. In this way, parents become initial agents showing social acceptance of drinking and may convey permissiveness of alcohol use thereby facilitating the initiation of drinking at an early age [17]. Thus, in Latin America, the risk of problematic alcohol use among youth experiencing a period of transition (e.g., the start of university) is significantly associated to their perception of parental drinking [7]. We also found that students’ drinking patterns resemble their parents’ drinking patterns when they are not at the parental home. It could be the case that those who moved out of the parental home may spend the weekends at their parents’ home and drink together with their parents, yet students may have already picked up their parents’ drinking habits and reenact them outside of the parental home [25]. Betancourth-Zambrano and colleagues [10] also found that Colombian students’ preferred occasion for consuming alcohol is celebrations with friends and peers, which indicates that alcohol consumption may primarily occur outside of the parental home. As living with parents has been often reported to serve as a protective barrier against alcohol use for university students [26], future studies among Colombian students should assess parental rules towards alcohol use inside and outside of the household to develop more comprehensive inferences. Furthermore, students did not behave—nor expected to behave—similarly to their parents regarding physical activity. As physical activity may reflect different behavior for parents than for students (e.g., physical activity related to the parents’ occupation rather than to sports), one would expect less similarity in physical activity levels because doing sports together or working together might only refer to a limited number of families and amount of time. Physical activity may also refer to times when students are not together with their parents (e.g., time with peers). In contrast, if students live with their parents, it is likely that joint meals and drinking occurs regularly simply because of the shared household. As such, joint eating and drinking may be the rule if co-residence exists, but joint physical activity may usually not be.

Third, regarding the partner, students’ current number of drinks consumed per month as well as current and expected binge drinking was significantly associated to the alcohol use of their partner. Moreover, perceived efforts from the partner to encourage the intake of alcohol moderated the association of students’ current and expected binge drinking with partners’ alcohol use. Our findings corroborate extensive evidence postulating that partners significantly influence each other’s alcohol consumption [27]. Thus, first semester students seem to binge drink together with their partner and to be influenced by a partner’s encouragement to drink.

Finally, regarding peers, a significant correlation was found regarding participants’ expected physical activity and their peers’ physical activity, which supports findings among Colombian students by Arango-Paternina and colleagues [11]. Nonetheless, students seem to not show many health behaviors similar to their peers. In Colombia, the percentage of students that live with their parents is much higher compared to those that live with their peers. This might mean that students spend less time with their peers and therefore have fewer opportunities to show health behaviors similar to their peers, which could explain the nonsignificant results. Alternatively, as the peer group is an umbrella term covering several affiliations to broad social groups, it could be that close friendships rather than the general peer group may have a stronger influence on students’ behaviors [28]. Another possible explanation may be that during the first months at university, an individual’s peer group is also in transition (from former classmates at high school to the students at university), which could temporarily reduce the impact of peers. Nonetheless, students who spend more time with their peers reported consuming a higher number of drinks in a month. We found a robust effect that remained significant even in the last step of the analysis corroborating the general consensus in the literature that spending more time with peers is related to more drinks consumed in a month among university students [9,10]. As such, time with peers is strongly associated with heavy drinking among first semester students in Colombia and may also be associated to binge drinking, although this association did not reach the 5% significance level in the present study; however, this requires further examination in future studies. Moreover, our results indicate that students who spend more time with their peers show healthier physical activity levels. Peers play an important role in many kinds of physical activities (e.g., team sports) and social motives often influence these activities [29], which may explain the observed positive association of time with peers and physical activity. Spending time with peers may also contribute to youths’ healthy behavior by providing the opportunity to develop social skills and autonomy, and to increase self-esteem [30]. Moreover, we speculate that participants might engage in physical activity together with their peers. Alternatively, as participants may not live close to and most do not live with their peers, they may need to engage in physical activity (e.g., walk or cycle) to meet peers.

### 4.1. Limitations and Future Research

Potential limitations should be considered when interpreting and generalizing the present results. First, we did not collect data from students below 18 years of age (because getting the parental permission for the participation of younger students would be challenging, particularly when students moved out of the parental home to attend university). Thus, it needs to be tested whether similar results would be found in younger students. Second, we could not analyze the moderating effects of living with a partner and with peers as the majority of participants reported living with their parents. Future investigations may recruit a higher number of participants who moved out of their parents’ home to assess the influence of living arrangement on first-semester students’ health behaviors. Third, we did not assess change of health behavior throughout the first semester, which may have provided additional novel findings for Colombian universities and interventionists. Future studies among university students in Colombia could assess changes in health behaviors from the beginning of the first semester and throughout different semesters to develop a comprehensive understanding of health behavior change among university students, factors that influence these changes, and possibilities for prevention. Finally, as we only assessed time with peers in general, it might be interesting to assess whether time spent with peers during the week or during the weekend promotes higher alcohol intake among first-semester students in Colombia, in order to advise parents, by means of intervention, to limit the time that their adult children spend with peers during specific times of the week.

### 4.2. Conclusions

Notwithstanding these limitations, with the present findings we conclude that in Colombia, parents appear to play a significant role in their offspring’s health behaviors during their first semester at university, particularly regarding food consumption and alcohol use. Moreover, drinking among students is strongly related to their partner’s drinking behavior, especially concerning binge drinking, which is reinforced by the partner’s motivation to consume alcohol. Furthermore, time spent with peers was strongly related to heavy drinking among students. Parental monitoring as well as limiting unhealthy activities that students do with their peers should be more strongly encouraged through interventional efforts, particularly in more collectivistic cultures where parents tend to have stronger influences on their offspring’s behavior compared to Western countries [31]. It is also advisable that parents limit their own alcohol use at home as their offspring are strongly influenced by this parental behavior. Interventions should also inform parents of their influence on their offspring’s food consumption and provide support as to how to promote the consumption of healthy food. Lastly, interventions could place a stronger focus on the partner’s influence on drinking by, for instance, demonstrating that drinking is not needed to enjoy time together.

## Figures and Tables

**Table 1:** Associations of living with parents and parental health behavior with students’ current and expected health behavior (Multiple regression analysis).

	Participant HB: Current Food Consumption			Participant HB: Current Physical Activity			Participant HB: Current # of Drinks Consumed Monthly			Participant HB: Current Binge Drinking		
	*B*	*SE B*	β	*B*	*SE B*	β	*B*	*SE B*	β	*B*	*SE B*	β
Parents’ HB	0.82	0.32	0.18 **	1.25	0.62	0.15 *	1.59	0.60	0.19 *	0.30	0.07	0.29 **
Living with Parents	−1.06	0.87	−0.14	3.31	1.44	0.23 *	2.37	1.39	0.15	0.59	0.16	0.29 **
Parents’ HB x Living with Parents	−0.38	0.68	−0.07	−1.10	1.21	−0.09	2.51	1.21	0.18 *	0.47	0.14	0.27 **
Δ*R*² Step 1		0.04 *			0.04 *			0.05 *			0.14 **	
Δ*R*² Step 2		0.01			0.01			0.02 *			0.05 **	
*R*² Total		0.05 *			0.05 *			0.07 *			0.19 **	
	**Participant HB: Expected Food Consumption**			**Participant HB: Expected Physical Activity**			**Participant HB: Expected # of Drinks Consumed Monthly**			**Participant HB: Expected Binge Drinking**		
	***B***	***SE B***	**β**	***B***	***SE B***	**β**	***B***	***SE B***	**β**	***B***	***SE B***	**β**
Parents’ HB	0.40	0.35	0.08	−0.46	0.62	−0.01	1.80	0.67	0.19 *	0.21	0.07	0.22 **
Living with Parents	−2.46	0.94	−0.31 **	2.08	1.46	0.14	4.17	1.55	0.23 *	0.50	0.15	0.27 **
Parents’ HB x Living with Parents	−1.71	0.74	−0.27 ^*^	0.01	1.22	0.01	2.52	1.36	0.16	0.42	0.13	0.26^**^
Δ*R*² Step 1		0.02			0.03			0.07			0.09 **	
Δ*R*² Step 2		0.03 *			0.01			0.01 *			0.05 **	
*R*² Total		0.05 *			0.04			0.08 *			0.14 **	

Note. HB = health behavior. *B* = unstandardized regression coefficient. *SE* = standard error. β = standardized regression coefficient. Step 1 assessed main effects and Step 2 the interaction effect. Living with parents was represented as a dummy variable. Parents’ HB refers to the corresponding participant behavior. For the interaction, parental HB and co-residence with parents were centered at their means. * *p* < 0.05, ** *p* < 0.01.

**Table 2:** Associations of social ties’ efforts to motivate unhealthy behaviors and social ties’ health behavior with students’ current health behavior (Multiple regression analysis).

	Participant HB: Current Food Consumption			Participant HB: Current Physical Activity			Participant HB: Current # of Drinks Consumed Monthly			Participant HB: Current Binge Drinking		
	*B*	*SE B*	β	*B*	*SE B*	β	*B*	*SE B*	β	*B*	*SE B*	β
Parents’ HB	−0.10	0.64	−0.02	1.46	2.06	0.18	2.19	1.62	0.26	0.42	0.19	0.40 *
Parents’ Motivation UB	0.95	0.39	0.26 *	0.26	0.80	0.03	−2.47	0.71	−0.28^*^	−0.33	0.09	−0.30 *
Parents’ HB x Parents’ Motivation UB	0.59	0.32	0.32	−0.20	0.76	−0.07	−0.39	0.62	−0.13	−0.06	0.07	−0.15
Δ*R*² Step 1		0.04 *			0.01			0.11 **			0.18 **	
Δ*R*² Step 2		0.02 *			0.00			0.01			0.01	
*R*² Total		0.06 *			0.01			0.12 **			0.19 **	
Partners’ HB	0.18	1.39	0.06	−0.60	2.97	−0.08	9.38	3.86	0.93 *	1.27	0.37	1.15 *
Partners’ Motivation UB	−0.83	0.60	−0.023	0.63	1.04	0.09	−3.00	1.35	−0.31 *	−0.40	0.13	−0.37 *
Partners’ HB x Partners’ Motivation UB	−0.34	0.66	−0.18	0.74	1.18	0.25	−2.58	1.54	−0.68	−0.32	0.15	−0.78 *
Δ*R*² Step 1		0.04			0.02			0.19 *			0.35 **	
Δ*R*² Step 2		0.01			0.01			0.04			0.05 *	
*R*² Total		0.05			0.03			0.23 *			0.40 *	
Peers’ HB	0.90	0.87	0.19	0.87	1.37	0.11	−0.49	1.23	−0.06	−0.10	0.15	−0.09
Peers’ Motivation UB	−0.30	0.45	−0.07	0.01	0.71	0.00	−2.70	0.59	−0.35 **	−0.30	0.07	−0.31 **
Peers’ HB x Peers’ Motivation UB	−0.22	0.45	−.009	−0.04	0.62	−0.01	0.52	0.57	0.13	0.10	0.07	0.19
Δ*R*² Step 1		0.01			0.01			0.11 **			0.09 *	
Δ*R*² Step 2		0.01			0.00			0.01			0.01	
*R*² Total		0.02			0.01			0.13 **			0.10 *	

Note. HB/UB = healthy/unhealthy behavior. *B* = unstandardized regression coefficient. *SE* = standard error. β = standardized regression coefficient. Step 1 assessed main effects and Step 2 interaction effects. Social tie’s HB and motivation refer to the corresponding participant behavior. For the interaction, social ties’ HB and their efforts to motivate unhealthy behaviors were centered at their means. * *p* < 0.05, ** *p* < 0.01.

**Table 3:** Associations of time spent with peers and living with parents with students’ current health behavior (Hierarchical regression analysis).

	Participant HB: Current Food Consumption			Participant HB: Current Physical Activity			Participant HB: Current # of Drinks Consumed Monthly			Participant HB: Current Binge Drinking		
	*B*	*SE B*	β	*B*	*SE B*	β	*B*	*SE B*	β	*B*	*SE B*	β
Age	−0.43	0.24	−0.13	−0.69	0.48	−0.11	0.17	2.63	0.02	−0.03	0.01	−0.04
Time with Peers	0.07	0.04	0.15 *	−0.13	0.07	−0.14 **	0.20	0.08	0.20 *	0.02	0.01	0.14
Living with Parents	−0.76	0.54	−0.10	2.01	1.06	0.14	1.25	1.18	0.08	0.34	0.15	0.17 *
Time with Peers x Living with Parents	−0.10	0.07	−0.11	−0.16	0.14	−0.08	−0.02	0.16	−0.01	0.01	0.02	0.01
Δ*R*² Step 1		0.02			0.01			0.01			0.01	
Δ*R*² Step 2		0.03			0.04 **			0.04 *			0.04 *	
Δ*R*² Step 3		0.01			0.01			0.00			0.00	
*R*² Total		0.06			0.06 **			0.05 *			0.06 *	

Note. HB = health behavior. *B* = unstandardized regression coefficient. *SE* = standard error. β = standardized regression coefficient. Step 1 assessed main effects and Step 2 the interaction effect. Time with peers referred to number of hours per week. Time with peers and living with parents were centered at their means to compute their interaction. * *p* < 0.05, ** *p* < 0.01.

## Data Availability

Not applicable.

## Supplementary Materials

The following supporting information can be downloaded at: https://www.mdpi.com/article/10.3390/ijerph20075370/s1, Table S1: Demographic characteristics.

## Abbreviations

*N* = total number of respondents; *M* = mean; *SD* = standard deviation.
