# Supplementary material for: Health Behaviors of Colombian First-Semester University Students in Association with Behaviors of Close Social Ties, Living Arrangement, and Time Spent with Peers
Source: Int J Environ Res Public Health. 2023 Apr 3;20(7):5370. doi: 10.3390/ijerph20075370 (PMC10094430; doi:10.3390/ijerph20075370)
Supplement: Supplementary file 1 [file ijerph-20-05370-s001.zip › ijerph-2251221-supplementary .pdf]

Table S1. Demographic characteristics.

Demographic Characteristics		*n*	*M*	*SD*
**Age**			18.79	1.071
	18	102		
	19	51		
	20	14		
	21	18		
	22	4		
**Sex**			1.69	.465
	Male	59		
	Female	130		
**Partner**			1.68	.469
	Yes	61		
	No	128		
**Ethnicity**			1.05	.241
	Colombian	180		
	From Abroad	9		
**Moved Out**			1.67	.473
	Yes	63		
	No	126		
**Living**			2.23	1.898
	With parents	120		
	Shared apartment	17		
	Shared dorm	6		
	With partner	7		
	Alone	13		
	Other	26		

Note. *SD* = standard deviation, *M*= mean. *N* = 189
